# Clinical and laboratory predictors for prosthetic joint infection within the first postoperative days following primary total hip and knee arthroplasty

**DOI:** 10.1007/s00264-023-05891-x

**Published:** 2023-07-08

**Authors:** Peter Brumat, Blaž Mavčič, Izak Jurčić, Rihard Trebše

**Affiliations:** 1 Valdoltra Orthopaedic Hospital, Jadranska cesta 31, 6280 Ankaran, Slovenia; 2 Faculty of Medicine, University of Ljubljana, Vrazov trg 2, 1000 Ljubljana, Slovenia; 3 Department of Orthopaedic Surgery, University Medical Centre Ljubljana, Zaloška 9, 1000 Ljubljana, Slovenia

**Keywords:** Prosthetic joint infection, PJI, Predictor, Total hip arthroplasty, Total knee arthroplasty

## Abstract

**Purpose:**

To identify clinical and laboratory predictors for low- and high-grade prosthetic joint infection (PJI) within the first postoperative days following primary total hip/knee arthroplasty (THA/TKA).

**Methods:**

Institutional bone and joint infection registry of a single osteoarticular infection referral centre was reviewed to identify all osteoarticular infections treated between 2011 and 2021. Among them were 152 consecutive PJI (63 acute high-grade PJI, 57 chronic high-grade PJI, and 32 low-grade PJI) who also had primary THA/TKA performed at the same institution, which were retrospectively analyzed with multivariate logistic regression and covariables.

**Results:**

For each additional day of wound discharge, persistent wound drainage (PWD) predicted PJI in the acute high-grade PJI group with odds ratio (OR) 39.4 (*p* = 0.000, 95%CI 1.171–1.661), in the low-grade PJI group with OR 26.0 (*p* = 0.045, 95%CI 1.005–1.579), but not in the chronic high-grade PJI group (OR 16.6, *p* = 0.142, 95%CI 0.950–1.432). The leukocyte count product of pre-surgery and POD2 >100 predicted PJI in the acute high-grade PJI group (OR 2.1, *p* = 0.025, 95%CI 1.003–1.039) and in the chronic high-grade PJI group (OR 2.0, *p* = 0.018, 95%CI 1.003–1.036). Similar trend was also seen in the low-grade PJI group, but was not statistically significant (OR 2.3, *p* = 0.061, 95%CI 0.999–1.048).

**Conclusions:**

The most optimal threshold value for predicting PJI was observed only in the acute high-grade PJI group, where PWD >three days after index surgery yielded 62.9% sensitivity and 90.6% specificity, whereby the leukocyte count product of pre-surgery and POD2 >100 showed 96.9% specificity. Glucose, erythrocytes, hemoglobin, thrombocytes, and CRP showed no significant value in this regard.

## Introduction

Prosthetic joint infection (PJI) is a major and devastating complication of joint arthroplasty, bearing a substantial burden to both the individual and society in terms of morbidity, mortality, and health care expenditure [1, 2]. Although the prevention of PJI is becoming more effective, the number of total arthroplasties in patients with increasing comorbidities continues to rise, and the total number of diagnosed and managed PJIs is expected to rise accordingly [1]. Acute and chronic high-grade PJIs, which frequently present as a life- or limb-threatening condition requiring prompt diagnostics and expedited treatment, are usually diagnosed in a straightforward manner. However, the diagnostics and management of low-grade PJIs remain intricate because of their often vague and unspecific clinical presentation [2]. Besides, PJI – colonization may be completely asymptomatic [2, 3]. The awareness of the problem of low-grade PJI has been increasingly recognized [2]. In addition to perspicuous PJIs, there is a significant although unidentified figure of septic failures concealed within aseptic failures [2, 4, 5]. Recent studies suggest there are up to 20% of low-grade PJI within presumed aseptic failures, whereby numerous early failures after revision arthroplasties may correlate with this overlooked category of PJI [2, 4, 6–8].

The appropriate evaluation and interpretation of diagnostic investigations are paramount in the management of PJI, especially for frequently unrecognized low-grade PJI, because inappropriate treatment of an unrecognized PJI usually ends with unacceptable and sometimes catastrophic results [2]. In the last decade, at least five different definitions of PJI have been proposed, each one with intrinsic limitations [9]. Despite several different PJI criteria, the evaluation and the role of potential clinical and laboratory parameters in predicting PJI within the first postoperative days after the index procedure is still lacking [9–15].

The aim of this study was the identification and assessment of potential laboratory (preoperative and on the second postoperative day (POD2)) and postoperative clinical parameters for predicting both low-grade and high-grade PJI in the first postoperative days following primary total hip/knee arthroplasty (THA/TKA).

## Materials and methods

### Study population and PJI criteria

An observational case-controlled study was performed in a single osteoarticular infection referral center. The institutional bone and joint infection registry was reviewed to identify all 715 surgically treated osteoarticular infections at our institution between 2011 and 2021. Among them, we performed a retrospective analysis of prospectively collected data on 152 consecutive PJI (32 low-grade PJI, 63 acute high-grade PJI, and 57 chronic high-grade PJI) who also had index surgery – primary THA/TKA performed at the same institution (out of the total 19096 primary THA/TKAs performed at our institution in the same time period).

All the PJIs at our institution are systematically managed by the dedicated Bone and Joint Infection Unit. We followed the institutional PJI criteria [2], which were based on the definition by Zimmerli et al. [16], until 2017, when we reached a consensus to switch to the EBJIS criteria [11]. We classified the PJIs as early or late acute based on the high degree of inflammation and severity of symptoms at presentation, timing after the index operation, and duration of symptoms. A PJI within the first month after the index operation was considered early, while a symptomatic PJI lasting more than four weeks was classified as chronic PJI, with a variable degree of inflammation and severity of disease progression.

There is no clear definition of “low-grade PJI” in the literature. For the purpose of this study, we defined it as a subtype of chronic, criteria-confirmed PJI, characterized by lasting, non-progressive (or oscillating) low-grade symptoms, primarily limited to pain and/or early loosening, associated with negative or low inflammatory parameters that do not show progressive or oscillating patterns.

### Control group

The size of the non-infected control group population was predetermined in a ratio of at least 1:1 (or more) with respect to the largest group of PJI subgroup and in a ratio of at least 2:1 with respect to the smallest PJI subgroup (among three PJI subgroups) and randomly selected by a single independent investigator, who was blinded to the study protocol. Inclusion criteria for the non-infected control group population were as follows: index surgery performed at our institution in the same period between 2011 and 2021, ≥two years of follow-up after primary THA or TKA without PJI or aseptic loosening diagnosis, patient’s age at the time of surgery ≥65 years, BMI at the time of surgery ≥25, ASA at the time of surgery ≥2, with equal share between THA and TKA, and equal share of both genders within the control group.

### Investigated parameters

The investigated demographic and surgeon-dependent variables were age (years), sex (male/female), BMI, cancer (yes/no), diabetes (yes/no), inflammatory joint disease (yes/no), tobacco use, operated joint (hip/knee), cemented implant (yes/no), ASA score (1, 2, 3, or 4), surgery duration (minutes), intra-articular tranexamic acid (TXA) application (yes/no), and surgical drain use (days). The investigated perioperative laboratory parameters (preoperative and on the POD2) were glucose level, erythrocyte count, hemoglobin level, thrombocyte count, leukocyte count, and C-reactive protein (CRP) level. The investigated postoperative clinical parameters were human body temperature (HBT) (degrees Celsius), measurement of pain intensity with VAS (0–10), frequency of wound dressing change (days), and persistent wound drainage (PWD) (days). Surgeon-dependent clinical parameters (frequency of wound dressing change, TXA application, surgical drain use) were treated as confounding variables.

### Statistical analysis

Tobacco use, HBT, measurement of pain intensity with VAS, and frequency of wound dressing change were excluded from the further analysis due to lack of data.

All other investigated laboratory and clinical parameters were then separately compared between (1) acute high-grade PJI and non-infected control group, (2) low-grade PJI and non-infected control group, and (3) chronic high-grade PJI and non-infected control group. The Mann-Whitney test was performed for continuous numeric variables and the Chi-square test for categorical variables.

The impact of laboratory and clinical parameters on PJI risk in the first postoperative days following primary THA/TKA was evaluated with multivariate analysis for (1) acute high-grade PJI vs. non-infected control group, (2) low-grade PJI vs. non-infected control group, and (3) chronic high-grade PJI vs. non-infected control group. Each multivariate model also included surgeon-dependent confounding variables (TXA application, surgical drain use). Furthermore, each laboratory parameter was systematically analyzed in a multivariate model for six different relations separately (preoperative parameter value, value on POD2, sum of preoperative and POD2 values, difference between preoperative and POD2 values, quotient of preoperative and POD2 values, and product of preoperative and POD2 values), surgeon-dependent variables, and PWD as the only predictive clinical parameter.

Sensitivity and specificity for PWD was further analyzed for each PJI group for the first seven postoperative days following primary THA/TKA and for the leukocyte product of preoperative and POD2 values for the threshold of 70, 80, 90, 100, 110, 120, 130, and 140.

Statistical analyses were performed using SPSS version 25.0 (IBM, Armonk, NY, USA). *P* < 0.05 was considered significant.

## Results

There was an uneven distribution of demographic parameters in the investigated PJI groups, with TKA preponderance in the low-grade PJI group, whereby the patients were less likely to get TXA during surgery. The patients in the acute high-grade PJI group had higher ASA scores and were more likely to get TXA; those in the chronic high-grade PJI group were mostly male and had a diagnosis of diabetes, and the patients in both acute and chronic high-grade PJI groups had significantly higher BMI. Patients in both low-grade and high-grade chronic PJI groups had surgical drains applied for longer duration (Table 1). The size of the non-infected control group population (*N* = 64) was equivalent to the largest investigated group (63 acute high-grade PJI) and matched 2:1 to the low-grade PJI group (*N* = 32).

**Table 1:** Comparison of average values of demographic, surgeon dependable, and clinical parameters

	Acute high-grade PJI group vs. non-infected control group (*N* = 63)	Low-grade PJI group vs. non-infected control group (*N* = 32)	Chronic high-grade PJI group vs. non-infected control group (*N* = 57)	Non-infected control group (*N* = 64)
Age (years)	68.37, *p* = 0.793	66.28, *p* = 0.180	65.78, *p* = 0.088	69.81
Sex (0 = female, 1 = male)	0.44, *p* = 0.531	0.50, *p* = 1.000	0.72, *p* = 0.014	0.50
BMI (kg/m^2^)	34.03, *p* = 0.000	31.49, *p* = 0.147	32.45, *p* = 0.002	29.86
Cancer (1 = yes, 0 = no)	0.12, *p* = 0.821	0.15, *p* = 0.838	0.11, *p* = 0.556	0.15
Diabetes (1 = yes, 0 = no)	0.21, *p* = 0.233	0.18, *p* = 0.838	0.32, *p* = 0.021	0.15
Inflammatory joint disease (yes/no)	0.15, *p* = 0.785	0.06, *p* = 0.551	0.05, *p* = 0.634	0.08
Operated joint (1 = hip, 2 = knee)	1.50, *p* = 0.788	1.79, *p* = 0.008	1.65, *p* = 0.098	1.50
Cemented implant (1 = yes, 0 = no)	0.48, *p* = 0.184	0.76, *p* = 0.099	0.63, *p* = 0.549	0.58
ASA score (1–4)	2.71, *p* = 0.006	2.44, *p* = 0.528	2.6, *p* = 0.108	2.40
Surgery duration (minutes)	81.94, *p* = 0.573	74.44, *p* = 0.802	82.11, *p* = 0.198	75.00
Intraarticular TXA application (1 = yes, 0 = no)	0.36, *p* = 0.041	0.21, *p* = 0.002	0.35, *p* = 0.078	0.28
Surgical drain use (days)	0.62, *p* = 0.053	0.88, *p* = 0.002	0.83, *p* = 0.002	0.40
PWD (days)	6.69, *p* = 0.000	2.91, *p* = 0.015	3.31, *p* = 0.011	1.28

Compared to the non-infected control group, PWD predicted PJI in the first postoperative days following THA/TKA in the acute high-grade PJI group with an odds ratio (OR) 39.4 (*p* = 0.000, 95%CI 1.171–1.661) for each additional day of wound discharge, and in the low-grade PJI group with OR 26.0 (*p* = 0.045, 95%CI 1.005–1.579) for each additional day of wound discharge, whereas this trend was not statistically significant in the chronic high-grade PJI group (OR 16.6, *p* = 0.142, 95%CI 0.950–1.432).

After systematic analysis and exclusion of surgeon-dependant variables, only the leukocyte count product of pre-surgery and POD2 >100 of the investigated laboratory parameters predicted PJI in the first postoperative days following THA/TKA in the acute high-grade PJI group (OR 2.1, *p* = 0.025, 95%CI 1.003–1.039), and in the chronic high-grade PJI group (OR 2.0, *p* = 0.018, 95%CI 1.003–1.036). Similar trend was also seen in the low-grade PJI group, but was not statistically significant (OR 2.3, *p* = 0.061, 95%CI 0.999–1.048). All other investigated laboratory parameters (glucose level, erythrocyte count, haemoglobin level, thrombocyte count, CRP) showed no significant value in the prediction of PJI within the first postoperative days following THA/THA.

The most optimal threshold value for predicting PJI based on PWD within the first postoperative days following THA/THA was observed only in the acute high-grade PJI group, where PWD of more than three days after index surgery yielded 62.9% sensitivity and 90.6% specificity (Table 2), whereby the leukocyte count product of pre-surgery and POD2 >100 showed 96.9% specificity (Table 3).

**Table 2:** Sensitivity and specificity for each PJI group

		PWD >0	PWD >1	PWD >2	PWD >3	PWD >4	PWD >5	PWD >6	PWD >7
Acute high-grade PJI group	Cases (#)	43	42	41	39	35	31	27	21
	Sensitivity (%)	69,4	67,7	66,1	62,9	56,5	50,0	43,5	33,9
Low-grade PJI group	Cases (#)	17	17	16	11	8	7	5	3
	Sensitivity (%)	56,7	56,7	53,3	36,7	26,7	23,3	16,7	10,0
Chronic high-grade PJI group	Cases (#)	29	28	24	21	18	16	11	7
	Sensitivity (%)	53,7	51,9	44,4	38,9	33,3	29,6	20,4	13,0
Non-infected control group	Cases (#)	26	19	14	6	5	4	3	1
	Specificity (%)	59,4	70,3	78,1	90,6	92,2	93,8	95,3	98,4

*PWD * persistent wound drainage, *PJI * prosthetic joint infection

**Table 3:** Sensitivity and specificity for each PJI group

		LKC 0-2 >70	LKC 0-2 >80	LKC 0-2 >90	LKC 0-2 >100	LKC 0-2 >110	LKC 0-2 >120	LKC 0-2 >130	LKC 0-2 >140
Acute high-grade PJI group	Cases (#)	29	21	14	11	10	6	5	0
	Sensitivity (%)	46,8	33,9	22,6	17,7	16,1	9,7	8,1	0,0
Low-grade PJI group	Cases (#)	12	8	4	4	3	1	0	0
	Sensitivity (%)	40,0	26,7	13,3	13,3	10,0	3,3	0,0	0,0
Chronic high-grade PJI group	Cases (#)	25	17	14	12	8	7	7	6
	Sensitivity (%)	46,3	31,5	25,9	22,2	14,8	13,0	13,0	11,1
Non-infected control group	Cases (#)	18	12	7	2	1	1	1	0
	Specificity (%)	71,9	81,3	89,1	96,9	98,4	98,4	98,4	100,0

*LKC 0-2 * leukocyte count product of pre-surgery and 2nd postoperative day, *PJI* prosthetic joint infection

## Discussion

PJI has a wide variety and severity of clinical presentations, whereby most of the PJIs manifest with signs and symptoms between the two extremes of acute, high-grade, and subclinical low-grade or even asymptomatic disease [2, 9]. A wide and heterogeneous set of PJI criteria, scoring systems, and reference values exist, and novel PJI markers and diagnostic tools are continuously discovered [9]. However, the importance of the clinical and laboratory parameters for suggesting an already ongoing but still unremarkable PJI within the first postoperative days following primary THA/TKA has not yet been evaluated. Particularly, the potential value of clinical and laboratory parameters for predicting PJI that will later manifest with low-grade symptoms might actually not differ in the early postoperative course from a postoperative course ending with a normally functioning aseptic THA/TKA [2]. This study aimed to identify these clinical and laboratory parameters in the early postoperative period that may predict later development of clinically important PJI, including low-grade PJI. The analysis revealed that the identified threshold value was specifically applicable to the acute high-grade PJI group, whereby PWD more than three days after the index surgery exhibited a sensitivity of 62.9% and a specificity of 90.6%. Moreover, a leukocyte count product exceeding 100, calculated by multiplying the pre-surgery leukocyte count with the leukocyte count on POD2, demonstrated a specificity of 96.9%.

Numerous risk factors are associated with a higher probability of PJI after total joint arthroplasty, namely internistic comorbidities, sex, higher BMI, tobacco use, steroids, high ASA grade, prolonged operative time, the use of non-antibiotic-laded cement, and previous joint surgery or infection [17–20]. The association of such risk factors with a higher probability of PJI was also shown in our study. However, for our study, these were confounding factors as our focus was not solely on the risk of infection itself, but rather on early signs for detecting PJI in the first postoperative days following THA/TKA.

CRP is widely used as a marker to screen for inflammatory complications, with the postoperative course at its maximum on the third postoperative day [21]. The clinical relevance of CRP profile for the detection of a future clinical PJI in the first postoperative days after THA and TKA for detecting PJI remains questionable [21, 22], as also in our study, we did not prove the importance of CRP in predicting PJI in the first few postoperative days.

With a recommendation against performing total joint arthroplasty on a patient who has received an intraarticular corticosteroid injection within three months, the current evidence suggests ipsilateral intra-articular corticosteroid injections within three months before arthroplasty are associated with an increased risk of PJI during subsequent joint arthroplasty [23], supporting our institution’s policy to postpone the elective arthroplasty at least for three months after such procedure. Thus, we did not include this parameter in our analysis.

Administration of TXA on the day of surgery in THA/TKA is associated with a statistically significant diminished risk of delayed PJI [20, 24]. Our institution’s established policy is to administer a combination of intravenous and intra-articular TXA during THA/TKA, although some of the staff surgeons do not concur with its intra-articular administration, which explains why we treated this factor in our analysis as a surgeon-dependent variable, similarly as the surgical drain use. Even after excluding the impact of these two confounding factors, the predictive value of PWD and the leukocyte product of preoperative and POD2 values remained significant.

A PWD is a well-known complication following THA/TKA and is a recognized risk factor for PJI [25]. It is, however, unclear whether PWD is the cause or the consequence of a PJI. Despite many definitions for PWD having been proposed, a validated description remains elusive, and evidence-based clinical guidelines for the diagnosis and treatment of PWD in TJA are still lacking [25]. Each extra day of PWD carries a high additional risk of wound infection after THA/TKA [25]. Drainage continuing after 72 h may arise from fat necrosis sustained during surgery, dissolving haematoma from poor haemostasis, or fluid from a deep capsular defect, and must be considered potentially infectious [25, 26]. According to the proceedings of international consensus on orthopedic infections, the suggested definition of persistent wound drainage is any continued fluid extrusion from the operative site occurring beyond 72 h from index surgery [25, 27]. However, there is no hard evidence when the surgical intervention for PWD is justified. Clinical signs and serological tests can be helpful in the diagnosis of developing infection, whereas surgical treatment is advised when wound drainage persists for more than five to seven days [28]. However, early interventions with stitches still carry a high bacterial burden, as the skin cannot be appropriately sterilized and may result in a PJI. A nonsurgical protocol investigating patients with PWD beyond postoperative day #5 has also been demonstrated as a successful management modality of post-arthroplasty wound drainage [29]. With the identification of modifiable risk factors of PWD, a similar study by Shahi et al. signified that a seven day time limit for conservative care is appropriate for the treatment of PWD [30]. The results of our study suggest it may not be worthwhile to search for an ideal cutoff value, but rather, other clinical and/or laboratory parameters should be considered as well. In this regard, the leukocyte count product of preoperative and POD2 can improve specificity but not sensitivity.

## Conclusions

Following primary THA/TKA, PWD may predict acute high-grade and low-grade PJI in the first postoperative days following primary THA/TK, whereby the dynamic of serum leukocyte count in the first two postoperative days further implies PJI. Among the laboratory parameters in the early postoperative days, it demonstrated to be the only one that can serve as a tool for decision-making regarding early PJI management. It may not be useful in terms of sensitivity (detecting infected cases), but it exhibits a high level of specificity (excluding false positives among non-infected cases) to prevent unnecessary revisions. Glucose level, erythrocyte count, haemoglobin level, thrombocyte count, and CRP were not shown to be associated with increased incidence of later development of a PJI. This may, in turn, suggest the inclusion of PWD in the PJI diagnostic criteria.

## Data Availability

Raw data was generated at the Valdoltra Orthopaedic Hospital, Ankaran, Slovenia. Derived anonymized data supporting the findings of this study are available from the corresponding author upon request.
